# The Medium and the Message: Fitting Sound Health Promotion Methodology Into 160 Characters

**DOI:** 10.2196/mhealth.3888

**Published:** 2014-11-03

**Authors:** Megan S C Lim, Cassandra Wright, Margaret E Hellard

**Affiliations:** ^1^Burnet InstituteCentre for Population HealthMelbourneAustralia; ^2^Monash UniversitySchool of Population Health and Preventive MedicineMelbourneAustralia

**Keywords:** text messaging, mobile phone, health promotion, program evaluation

## Abstract

Text messaging health promotion projects continue to proliferate due to their relative low-cost, simplicity, non-intrusiveness, and proven effectiveness in several randomized controlled trials. In these past trials, participants have typically been recruited through traditional means, received the text messaging intervention, and then completed evaluation. In this issue of the *Journal of Medical Internet Research*, Sheoran et al have demonstrated how use of text messaging alone can be a feasible method for all three stages: recruitment, intervention, and evaluation. Use of text messages without any other modes of communication could be a key to population-level dissemination and wider uptake of health promotion messages. However, in the rush to utilize new technologies and in the brevity of 160 characters, it should not be forgotten that quality, rigour, and careful development remain essential in any health promotion practice.

## Introduction

There are now 96 mobile subscriptions for every 100 people on the planet [1], and text messaging (SMS, short message service) remains one of the most common uses of mobile phones, particularly for young people in the United States [2]. Consequently, text messaging is being increasingly used as a health promotion tool, as highlighted recently by Sheoran et al in the *Journal of Medical Internet Research* [3]. As technologies develop and projects in this field proliferate, it is vital that quality research continues to be conducted to maximize the quality of interventions, to refine the methods used, and to evaluate impact.

While the major benefit of text messaging interventions is their ability to reach people in a non-intrusive way, most trials published to date have bookended the text messaging intervention itself with traditional recruitment methodologies such as face-to-face or clinic-based recruitment and traditional evaluation methodologies such as focus group discussions or questionnaires [4-6]. While this might have been considered necessary initially to provide scientific rigour, the cost and effort of going through these methods negates the key benefits and simplicity of the technology. However, it is possible to use text messages as the sole method of communication in an intervention; to identify and recruit participants, to deliver the intervention, and to evaluate the project. *The Hookup* provides an example of a sexual health promotion project using text messaging in all of these three ways.

## Text Messaging to Recruit Participants

Text messaging health promotion and research efforts have often relied on starting with face-to-face recruitment [4]. However, this may not be necessary and means that some of the benefits of mobile-delivered health promotion or research are lost—in particular it is more costly, time-consuming and inconvenient for both researchers and participants. Instead, researchers could utilize text messaging to recruit people with whom they have never had any contact with in any other form.

One of the greatest advantages in using text messaging to recruit participants is the feasibility for population-wide dissemination and translation of research into practice; a much lower level of engagement is needed than for other contact methods. This may be particularly salient for young people; other evaluation methods such as postal mail and landline communication have limited reach for this demographic group and may recruit only the most motivated young people. Through mixing technologies and approaches, it may be possible to reach different people who do not normally engage in research. The *Hookup*’s recruitment approach was opt-in, via text messages [3]. They advertised the service widely at schools, adolescent health centers, and online but had no direct contact with potential participants. Other programs have attempted to attract users through in-person enrollment at festivals or street intercept, and use of mass media [5, 7-9].

Probably the most effective and efficient way to recruit participants via text messages is to partner with a telecommunications company or mobile phone provider. This allows individuals’ mobile phones to be accessed in large numbers from existing databases with very little effort [10,11]. However, as discussed in depth by Gold et al, this can be problematic when the priorities of the private company and the public health or research organization do not align [11]. A particular concern is that researchers become dependent on a third party (the private telecommunications company) to conduct their research; for example in one study 316 text message surveys were not delivered due to a mid-study block by a local mobile phone provider [12], and in another study the telecommunications company elected to censor the content of sexual health promotion messages [11]. Other important potential limitations to using text messaging to recruit participants are that engagement with the program may be lower, initial participation rates may be lower, and it may not be possible to validate participant characteristics (eg, age and gender) [11,13].

## Text Messaging as the Intervention

Sheoran et al report that *The Hookup* project was successful, with 90% of respondents reporting a positive change to their behavior such as testing for sexually transmitted infection (STI) or condom use [3]. This work supports the findings of several previous trials and reviews [4,5,14-17]. However, despite the success of text messaging interventions in sexual health promotion, there remains important research gaps in this area. While several studies have shown impact using text messaging, this does not mean that all text messaging programs will be effective; significant work is needed to develop effective content. As Ybarra et al, pointed out “many researchers seem to view text messaging as the intervention itself instead of simply a delivery mechanism… Like other interventions, however, the content is a central driver of the behavior change.”[18] There has been limited use of behavior change theory to guide message development in many previous studies [15]. The impact of important variables on the success of a project; including the timing, frequency, duration, tailoring, and interactivity of text messaging has not been fully explored [4,17]. Furthermore, while mHealth sexual health promotion interventions show great promise in developing countries [19], to date there are no reported RCTs in this setting.

## Text Messaging to Evaluate the Intervention

When evaluating a text messaging health promotion intervention, typical outcomes include process measures such as acceptability of the intervention and impact measures such as changes in knowledge and behaviors. Process measures can use a combination of data on number of subscribers, response rates and dropout rates, which should be collated throughout the project. Additionally, participants may be asked to report their opinions about the program generally and the content specifically. Impact measures can also utilize self-report data (eg, in the *Hookup* participants reported whether they had changed their behavior).

Common methods used previously to collect these self-report data include focus group discussions and surveys [5,8,20]. However, as Sheoran et al describe, in an intervention where the only contact researchers have with participants is via text messages this may not be feasible. Online evaluation is considerably simpler than 10 years ago now that many recipients can switch from a text message to an online survey instantly on a single device using Web-enabled mobile phones. However, this may bias participation to those who own smartphones and exclude those who choose only to engage with the program via text messaging. An alternative is to use text messages themselves to collect self-report questionnaire data.

Text messaging data collection in research is well established [12,21-24]. This method is effective, low-cost and useful, and achieves good response rates compared to other survey delivery methods [12,22,25,26]. The major limitation of text messaging data collection is the number of characters; the 160 character limit can make it difficult to provide sufficient detail to pose clear and valid questions. However, several studies have compared text messaging questionnaire responses to retrospective reports by more traditional survey methodologies and have demonstrated reliability over time in responses [12,22,25].

An additional benefit of text messaging data collection is that it allows the possibility of using frequent (eg, daily) real-time diary-style data collection. This may improve the reliability of data beyond retrospective questionnaires through decreasing recall bias and is particularly useful for rapidly fluctuating measures (eg, mood)[12,22,27,28]. One concern is that frequent prompts to complete data collection may influence behavior by serving as a reminder about the behavior in question; for example, one study’s participants reported that participating in a weekly text messaging sexual behavior survey made them want to have sex more often [22].

No matter how they are collected, self-report data are subject to certain biases and limitations [29]. Objective biological measures (eg, STI test results) would improve validity. Mobile technologies may be a pathway to achieving this; a consortium in the United Kingdom are working to develop mobile phone enabled point-of-care STI diagnostics for STI which link directly to mobile networks [30]. Other novel technological tools for evaluating the impact of a health promotion project include direct and objective mobile data collection via an app, wearable cameras, or other electronic devices such as accelerometers [31,32]. However, these sorts of devices may not be appropriate or acceptable for measuring sexual health related behaviors.

## Discussion

Text messaging interventions have demonstrated effectiveness in several trials, however, there is still much to learn about how to maximize their impact. As these programs are more broadly implemented, it is important to maintain rigour in development and evaluation. When moving beyond research into health promotion practice, the burden of poor funding and shortage of research-trained staff can prohibit the conduct of high quality evaluation via other methods. Thankfully as discussed above, the text messaging tool lends itself to practical and low-cost intervention delivery, recruitment, and evaluation.

Text messaging health promotion is an exciting area offering many opportunities in implementation and evaluation, but it is vital to ensure we are not leaving behind the theory and evidence that has been carefully developed and tested on other platforms. Even though a text message is only 160 characters long, it does not mean that work in this field requires less careful consideration. While text messaging is a highly promising tool, it is important to remember that text messaging itself is not the answer but only a mode of delivery. Whatever medium we use, we still need to be certain that we are recruiting the right people, delivering the right message, and measuring the right things to conduct successful and rigorous research and health promotion.

## References

[1] (2014). International Telecommunication Union.

[2] Duggan M, Rainie L (2012). Cell Phone Activities 2012.

[3] Sheoran B, Braun RA, Gaarde J, Levine DK (2014). The Hookup: Collaborative Evaluation of a Youth Sexual Health Program Using Short Message Service (SMS) Technology. J Med Internet Res.

[4] Fjeldsoe BS, Marshall AL, Miller YD (2009). Behavior change interventions delivered by mobile telephone short-message service. Am J Prev Med.

[5] Lim MS, Hocking JS, Aitken CK, Fairley CK, Jordan L, Lewis JA, Hellard ME (2012). Impact of text and email messaging on the sexual health of young people: a randomised controlled trial. J Epidemiol Community Health.

[6] de Tolly KM, Constant D (2014). Integrating mobile phones into medical abortion provision: intervention development, use, and lessons learned from a randomized controlled trial. JMIR Mhealth Uhealth.

[7] Buis LR, Hirzel L, Turske SA, Des Jardins TR, Yarandi H, Bondurant P (2013). Use of a text message program to raise type 2 diabetes risk awareness and promote health behavior change (part I): assessment of participant reach and adoption. J Med Internet Res.

[8] Gold J, Lim MS, Hocking JS, Keogh LA, Spelman T, Hellard ME (2011). Determining the impact of text messaging for sexual health promotion to young people. Sex Transm Dis.

[9] Lee HY, Koopmeiners JS, Rhee TG, Raveis VH, Ahluwalia JS (2014). Mobile phone text messaging intervention for cervical cancer screening: changes in knowledge and behavior pre-post intervention. J Med Internet Res.

[10] Chib A, Wilkin H, Ling LX, Hoefman B, Van Biejma H (2012). You have an important message! Evaluating the effectiveness of a text message HIV/AIDS campaign in Northwest Uganda. J Health Commun.

[11] Gold J, Hellard M, Lim M, Dixon H, Wakefield M, Aitken C (2012). Public-Private Partnerships for Health Promotion. American Journal of Health Education.

[12] Curran K, Mugo NR, Kurth A, Ngure K, Heffron R, Donnell D, Celum C, Baeten JM (2013). Daily short message service surveys to measure sexual behavior and pre-exposure prophylaxis use among Kenyan men and women. AIDS Behav.

[13] Kew S (2010). Text messaging: an innovative method of data collection in medical research. BMC Res Notes.

[14] Cole-Lewis H, Kershaw T (2010). Text messaging as a tool for behavior change in disease prevention and management. Epidemiol Rev.

[15] Free C, Phillips G, Galli L, Watson L, Felix L, Edwards P, Patel V, Haines A (2013). The effectiveness of mobile-health technology-based health behaviour change or disease management interventions for health care consumers: a systematic review. PLoS Med.

[16] Gold J, Aitken CK, Dixon HG, Lim MS, Gouillou M, Spelman T, Wakefield M, Hellard ME (2011). A randomised controlled trial using mobile advertising to promote safer sex and sun safety to young people. Health Educ Res.

[17] De Leon E, Fuentes LW, Cohen JE (2014). Characterizing periodic messaging interventions across health behaviors and media: systematic review. J Med Internet Res.

[18] Ybarra ML, Holtrop JS, Bağci Bosi AT, Emri S (2012). Design considerations in developing a text messaging program aimed at smoking cessation. J Med Internet Res.

[19] Vahdat HL, L'Engle KL, Plourde KF, Magaria L, Olawo A (2013). There are some questions you may not ask in a clinic: providing contraception information to young people in Kenya using SMS. Int J Gynaecol Obstet.

[20] Wilkins A, Mak DB (2007). . . . Sending out an SMS: an impact and outcome evaluation of the Western Australian Department of Health's 2005 chlamydia campaign. Health Promot J Austr.

[21] Whitford HM, Donnan PT, Symon AG, Kellett G, Monteith-Hodge E, Rauchhaus P, Wyatt JC (2012). Evaluating the reliability, validity, acceptability, and practicality of SMS text messaging as a tool to collect research data: results from the Feeding Your Baby project. J Am Med Inform Assoc.

[22] Lim MS, Sacks-Davis R, Aitken CK, Hocking JS, Hellard ME (2010). Randomised controlled trial of paper, online and SMS diaries for collecting sexual behaviour information from young people. J Epidemiol Community Health.

[23] Bexelius C, Merk H, Sandin S, Ekman A, Nyrén O, Kühlmann-Berenzon S, Linde A, Litton JE (2009). SMS versus telephone interviews for epidemiological data collection: feasibility study estimating influenza vaccination coverage in the Swedish population. Eur J Epidemiol.

[24] Lagerros YT, Sandin S, Bexelius C, Litton JE, Löf M (2012). Estimating physical activity using a cell phone questionnaire sent by means of short message service (SMS): a randomized population-based study. Eur J Epidemiol.

[25] Ybarra ML, Holtrop JS, Prescott TL, Strong D (2014). Process evaluation of a mHealth program: Lessons learned from Stop My Smoking USA, a text messaging-based smoking cessation program for young adults. Patient Educ Couns.

[26] Khosropour CM, Johnson BA, Ricca AV, Sullivan PS (2013). Enhancing retention of an Internet-based cohort study of men who have sex with men (MSM) via text messaging: randomized controlled trial. J Med Internet Res.

[27] Garcia C, Hardeman RR, Kwon G, Lando-King E, Zhang L, Genis T, Brady SS, Kinder E (2014). Teenagers and texting: use of a youth ecological momentary assessment system in trajectory health research with latina adolescents. JMIR Mhealth Uhealth.

[28] Schnall R, Okoniewski A, Tiase V, Low A, Rodriguez M, Kaplan S (2013). Using text messaging to assess adolescents' health information needs: an ecological momentary assessment. J Med Internet Res.

[29] Catania JA, Gibson DR, Chitwood DD, Coates TJ (1990). Methodological problems in AIDS behavioral research: influences on measurement error and participation bias in studies of sexual behavior. Psychol Bull.

[30] UKCRC translational infection research initiative consortium.

[31] Dalton AF, Ní Scanaill C, Carew S, Lyons D, OLaighin G (2007). A clinical evaluation of a remote mobility monitoring system based on SMS messaging. Conf Proc IEEE Eng Med Biol Soc.

[32] Gemming L, Doherty A, Kelly P, Utter J, Ni Mhurchu C (2013). Feasibility of a SenseCam-assisted 24-h recall to reduce under-reporting of energy intake. Eur J Clin Nutr.

